# S-100 Proteins: Basics and Applications as Biomarkers in Animals with Special Focus on Calgranulins (S100A8, A9, and A12)

**DOI:** 10.3390/biology12060881

**Published:** 2023-06-19

**Authors:** José Joaquín Cerón, Alba Ortín-Bustillo, María José López-Martínez, Silvia Martínez-Subiela, Peter David Eckersall, Fernando Tecles, Asta Tvarijonaviciute, Alberto Muñoz-Prieto

**Affiliations:** 1 Interdisciplinary Laboratory of Clinical Analysis (Interlab-UMU), Regional Campus of International Excellence ‘Campus Mare Nostrum’, University of Murcia, Campus de Espinardo s/n, 30100 Murcia, Spain; jjceron@um.es (J.J.C.); alba.ortinb@um.es (A.O.-B.); mariajose.lopez28@um.es (M.J.L.-M.); silviams@um.es (S.M.-S.); david.eckersall@glasgow.ac.uk (P.D.E.); ftecles@um.es (F.T.); alberto.munoz@um.es (A.M.-P.); 2 Institute of Biodiversity, Animal Health and Comparative Medicine, University of Glasgow, Garscube Estate, Glasgow G61 1QH, UK

**Keywords:** calgranulins, S100, calprotectin, biomarkers, inflammation

## Abstract

**Simple Summary:**

S100 proteins are a group of calcium-binding proteins which have a similar molecular mass and share similarities in their amino acid sequence. They are expressed in many tissues, and to date 25 different types of S100 proteins have been identified. They have a wide range of biological functions at both intracellular and extracellular levels. This review aims to provide updated information about S100 proteins and their use as biomarkers in veterinary science, with special emphasis on the family of calgranulins, which comprise a group of S100 proteins including S100A8 (calgranulin A), S100A9 (calgranulin B), and S100A12 (calgranulin C). The proteins SA100A8 and S100A9 can be linked, forming a heterodimer which is known as calprotectin. This review will help to improve basic knowledge of the S100 proteins as well their potential use as biomarkers in the veterinary field.

**Abstract:**

S100 proteins are a group of calcium-binding proteins which received this name because of their solubility in a 100% saturated solution of ammonium sulphate. They have a similar molecular mass of 10–12 KDa and share 25–65% similarity in their amino acid sequence. They are expressed in many tissues, and to date 25 different types of S100 proteins have been identified. This review aims to provide updated information about S100 proteins and their use as biomarkers in veterinary science, with special emphasis on the family of calgranulins that includes S100A8 (calgranulin A; myeloid-related protein 8, MRP8), S100A9 (calgranulin B; MRP14), and S100A12 (calgranulin C). The proteins SA100A8 and S100A9 can be linked, forming a heterodimer which is known as calprotectin. Calgranulins are related to the activation of inflammation and the immune system and increase in gastrointestinal diseases, inflammation and sepsis, immunomediated diseases, and obesity and endocrine disorders in different animal species. This review reflects the current knowledge about calgranulins in veterinary science, which should increase in the future to clarify their role in different diseases and potential as biomarkers and therapeutic targets, as well as the practical use of their measurement in non-invasive samples such as saliva or feces.

## 1. General Concepts and Functions of S100 Proteins and Objective of This Review

### 1.1. Definition

S100s are a group of calcium-binding proteins which received this name because of their solubility in a 100% saturated solution of ammonium sulphate at neutral pH [1]. All members of the S100 protein family have a similar molecular mass of 10–12 KDa, and they each share 25–65% similarity in their amino acid sequence [2]. They belong to the superfamily of the calcium-binding EF-hand proteins, the EF-hand being a helix-loop-helix structural domain or motif [3]. These proteins have charged amino acid residues, resulting in their affinity for divalent ions such as Ca^2+^, Zn^2+^, and Cu^2+^ [4]. Ca^2+^ binds to the EF-hand sites, while Zn^2+^ and Cu^2+^ bind to separate sites. They have the ability to form homodimers, heterodimers, and oligomeric assemblies which can have different physiological activities [5,6].

Although initially they were thought to be of neural origin since they were purified from the bovine brain, they are expressed in many tissues, and to date 25 different types of S100 proteins have been identified [2], and crystal structure forms of some of them have been described [7]. The main characteristics of each of the different S100s are summarized in Table 1.

### 1.2. Functions

S100 proteins have important roles and functions in live organisms at both intracellular and extracellular levels.

#### 1.2.1. Intracellular

S100 proteins have many intracellular roles through their interaction with several effector proteins within cells and their binding with Ca^2+^, Zn^2+^, and Cu^2+^. Therefore, they are involved in the regulation of multiple cellular processes such as contraction, motility, cell growth, division and differentiation, transcription, enzyme activation, and also in the structure of membranes, and dynamics of cytoskeleton constituents. In addition, they have a role in protection from oxidative cell damage and protein phosphorylation and secretion [85]. In many cases, S100 proteins function as Ca^2+^ sensor proteins, which on binding Ca^2+^, change their structure, and in this state they can interact with various target proteins changing their activity. The roles of Zn^2+^ and Cu^2+^ are not yet clear [86].

#### 1.2.2. Extracellular

In situations related to cell damage and/or activation, S100 proteins can be released into the extracellular space and promote various paracrine and autocrine functions such as inflammation, autoimmunity, and also cell proliferation and survival (including neuronal survival and extension) [85]. These roles would be related to the elevated S100 protein concentrations that are found in inflammatory processes, autoimmune diseases, neurodegeneration, and neoplasms.

### 1.3. Objective

This review aims to provide updated information about S100 proteins and their use as biomarkers in veterinary science, with special emphasis on the family of calgranulins, which comprise a group of S100 proteins including S100A8 (also named calgranulin A; myeloid-related protein 8, MRP8), S100A9 (calgranulin B; MRP14), and S100A12 (calgranulin C). The proteins SA100A8 and S100A9 can be linked, forming a heterodimer that is known as calprotectin and is described in humans as the most abundant form of these proteins in serum [87]. Therefore, the S100A8/S100A9 complex is also referred to as calprotectin or S100A8/A9 in this review. Although S100A8/A9 is released primarily as a dimer that has a proinflammatory effect, recently it has been described that, in some circumstances, it can form tetramers that can act as modulators protecting from overwhelming inflammation [88]. In addition to their presence in humans, some of these proteins have been specifically isolated in different animal species such as dogs [89] or pigs [90] but are only known to exist in vertebrate animals [86].

This review will be focused on calgranulins which are gaining interest because they are considered as specifically linked to innate immune functions and inflammation [91]. There are some differences between individual calgranulins:

- SA100A8 and A9 are released from granulocytes, monocytes, and macrophages, whereas SA100A12 appears more restricted to granulocytes [91,92].
- Cellular effects are mediated by binding of the SA100A8/A9 complex to pattern recognition receptors (PRRS) which are present in immune cells, such as toll-like receptors (TLRs) and receptors for advanced glycation end products (RAGE), but only RAGE receptors are bound by S100A12 [93]. Therefore, these proteins may activate endothelial and tissue cells differently according to the expression of the receptor [94]. Although these findings have been described in humans, in a recent report on dogs, it was observed that calprotectin and S100A12 are ligands for RAGE in the gastrointestinal tract [95].
- S100A12 does not exist in rodents [96].
- They can be involved in different physiological functions and mechanisms [97]. However, in studies of human serum [97,98] a high correlation has been found between the concentrations of S100A8/A9 and S100A12, and this could indicate a similar liberation pathway.

## 2. Mechanism of Action

**Figure 1:**
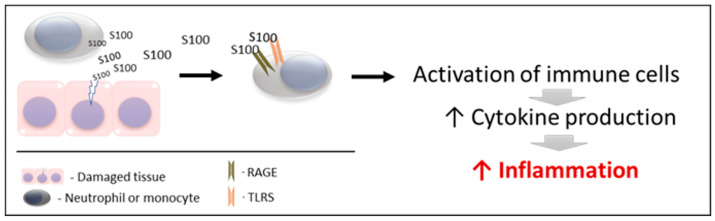
Schematic mechanism of action of S100 proteins. RAGE: receptors for advanced glycation end products. TLRs: toll-like receptors.

S100s and especially calgranulins mediate, among other processes, the inflammatory response, being considered important proinflammatory factors of innate immunity, which has a key role in host defense and in initiating inflammation. For a better understanding, two main phases can be differentiated in the mechanism of action of these proteins (Figure 1).

### 2.1. Action 1

S100 proteins are released by damaged and/or activated cells, under conditions of cell stress. Due to this mechanism of liberation, S100 proteins are considered to be “damage-associated molecular pattern proteins (DAMPs)”, “endokines”, or “alarmins” [91]; referring to compounds that, after any cell damage/stress and/or activation of immune cells (such as neutrophils and macrophages) are released to the extracellular space, where they play a key role in the regulation of several immune and inflammatory processes [99].

Interestingly, the three calgranulins do not have the structural elements required for secretion via the classical endoplasmic reticulum and Golgi-dependent secretory pathway. Therefore, they are released by cells by necrosis or cell death and also utilize an active cytoskeleton-dependent non-classical secretion that is used by some cytokines after cell activation [100].

It is interesting to point out that this mechanism is different from other mediators of innate immunity that are also used as biomarkers of inflammation and innate response such as the acute phase proteins, which are produced mainly by the liver mediated by interleukins, after an inflammatory stimulus [101]. This could explain why in some cases DAMPs can provide information on cell lesions that do not correlate with other inflammatory markers such as interleukins or acute phase proteins [94,102]. Therefore, acute phase proteins and calgranulins could be considered biomarkers that could provide complementary information about inflammation and innate immunity.

### 2.2. Action 2

The released S100 molecules of Action 1 are able to activate immune cells, promoting cytokine production and the inflammatory response. This is achieved by binding and interacting with the PRRS which are present in these cells (such as TLRs and RAGE). Therefore, they can interact and activate receptors similar to those activated by pathogen-associated molecular patterns (PAMPs) [103,104,105]. Recently, it has been demonstrated that S100 proteins can interact directly with cytokines, and such interactions can affect the functioning of these cytokines [106].

Although there is not any data about the half-life for all S100 proteins, the biological half-life of S100B is approximately 30 min, being eliminated mainly by the kidneys [85]. If this finding is similar to other S100 proteins, it can be stated that any persistent elevation of S100 serum levels would indicate their continuous release from affected tissues or activated cells. In addition, potential alterations in renal function could reduce their excretion and produce increases in these proteins.

## 3. Measurement

Usually, S100 proteins in humans are measured with immunological assays using specific antibodies and ELISA formats. These assays can be commercially available [87,107] or developed in-house [98]. In the case of calprotectin, in addition to ELISAs, it can be measured with automated turbidimetric immunoassays, obtainable from various suppliers and run on clinical biochemistry analyzers [108,109], with the advantages that the automation can have in clinical routine settings, such as higher precision and sample throughput.

These proteins can be measured in different sample types such as serum [110], feces [111], saliva [107,112], urine [113], or bronchoalveolar lavage fluid (BALF) [114]. As an example, in a report that studied serum, saliva, and urine in systemic lupus erythematosus in humans, the measurement of S100A8 showed good diagnostic ability in all sample types [87].

High-performance liquid chromatography coupled with electrospray ionization mass spectrometry has been used to simultaneously analyze several S100 proteins and their posttranslational modifications, complexes, and isoforms. This technique was able to identify in saliva different isoforms of S100 proteins, such as four isoforms of S100A8/S100A9 and two isoforms of S100A12 [115,116]. In addition, top-down and bottom-up proteomics detected S100A8 and A9 in different circulating complexes and proteoforms, which discriminated between survivors and non-survivors in septic shock patients [117].

In animals, species-specific assays have been used in different species such as dogs for the measurement of S100A8/A9 [102] and S100A12 [118] and cats for S100A12 [119]. In addition, a human turbidimetric immunoassay has been validated for the measurement of calprotectin in the saliva of pigs [120] and horses [121], and also in feces of dogs and cats [122] and pigs [123]. Therefore, there is the possibility of using heterologous immunoassays for the measurement of calprotectin, as used for other analytes such as acute phase proteins [124], providing the necessary analytical validation has been undertaken.

## 4. General Applications in Humans

In humans, calgranulins have been widely studied, and they have been described as biomarkers of different diseases and conditions. Various reviews can be found in the literature about these proteins and their clinical applications [45,85,125,126,127]. Overall, calgranulins have been evaluated in a variety of human diseases.

### 4.1. Gastrointestinal Disease

The extravasated neutrophils, which can be found in abundant number in the gastrointestinal system of patients with active inflammatory bowel disease (IBD), are a rich source of released S100A8/A9 which is detectable in feces. Therefore, calprotectin in feces has been used as a biomarker in these patients, being a disease activity indicator [128,129]. Measurement of S100A8/S100A9 in feces is considered a reliable method to distinguish IBD patients from those without chronic intestinal inflammation and it is, together with serum levels of S100A8/S100A9, useful in monitoring inflammation in patients with Crohn’s disease or ulcerative colitis [130]. In addition, S100A12 has been found to be massively expressed in inflamed tissue from patients with active IBD, and its serum concentrations correlate well with disease activity in individual patients [131].

### 4.2. Inflammation and Sepsis

After bacterial infection, neutrophils, macrophages, and monocytes intensely express and secrete calgranulins, with the role of modulating the inflammatory response, leading to the induction of inflammatory cytokines and also to the release of reactive oxygen species. Although still not totally clarified, S100A8 and S100A9 can have antibacterial potential due to their ability to bind Zn [4]. Of relevance is that deficiency of calprotectin has been related to the progress of pneumonia in *Staphylococcus aureus* infection in mice [132]. In addition, high concentrations of S100A9 have been involved in phagocyte hyperresponsiveness in sepsis and inflammatory conditions that can result in enhanced survival from septic shock [133]. However, there is some controversy to this, since other reports indicated that calgranulin can facilitate bacterial growth [134].

In sepsis, values of calprotectin were reported to be higher than in non-septic inflammation [135] and also correlate with the severity of the disease [136]. In addition, high plasma values of S100A8/A9 and S100A12 at admission can indicate a higher risk of death in septic shock patients [97]. In general, it is considered that calprotectin measurement allows early diagnosis of sepsis on admission of patients to intensive care units (ICU), being a tool that can aid timely sepsis management, reducing mortality rates and avoiding unnecessary antibiotic treatment, thus improving antibiotic stewardship [45].

### 4.3. Immunomediated Diseases

In systemic lupus erythematosus (SLE), there are increases in calprotectin that correlated with the SLE disease activity index [137]. Similarly, in rheumatoid arthritis (RA) and psoriatic arthritis serum concentrations of S100A8/S100A9 are related to the inflammatory activity of arthritis, being superior to other biomarkers such as C-reactive protein and erythrocyte sedimentation rate [138,139]. Additionally, S100A12 serum concentrations correlate with disease activity in RA [140], and calgranulins are also at high concentrations in synovial fluid in patients with this disease [91].

In addition, in dermatomyositis, polymyositis, and inclusion body myositis there is an association between S100A8 and S100A9 expression (possibly produced by the infiltration of macrophages) and degeneration of myofibers [141].

### 4.4. Obesity and Endocrine Disorders

The S100A8/A9 complex is implicated in the pathophysiology of obesity-promoting macrophage-based inflammation. In addition, serum levels of S100A8/A9 and S100A12 correlate with insulin resistance/type 2 diabetes, metabolic risk score, and fat cell size [142]. Additionally, calprotectin in type 2 diabetes is related to the degree of microvascular alteration at the glomerular and retinal bed, being a potential biomarker for microcirculatory defects associated with this disease [143].

### 4.5. Other Diseases

Other diseases in which calgranulins are increased are cardiovascular, neoplastic, skin, and neurological disorders including traumatic brain injuries and chronic neurodegenerative disorders such as Alzheimer’s disease [85,125,144]. Of further interest, S100A9 has been shown to be involved in CD 36 signaling in platelets, which is reported to be a key signal in arterial thrombosis, but not for physiologic hemostasis. Therefore, immunization of mice with a S100A9 vaccine resulted in long-term inhibition of thrombus formation through inhibition of increased S100A9/CD36 signaling, without risk of bleeding or adverse autoimmune responses [145].

Overall, in humans, S100s are considered an important group of both molecular key players and biomarkers in the etiology, progression, manifestation, and therapy of many disorders in which they have been studied [6]. Therefore, it can be stated that their study and analysis can provide information about physiopathology and also their use as possible biomarkers of disease activity and treatment monitoring in a high number of clinical disorders.

## 5. Gastrointestinal Disease in Animals

The use of calprotectin for the evaluation of gastrointestinal disease is the best-known and most frequent application of S100 proteins in veterinary medicine, having been used in several different animal species.

### 5.1. Dogs

In dogs with idiopathic IBD, fecal calprotectin increases and then decreases after treatment, with a significant correlation of r = 0.60 between fecal calprotectin and canine inflammatory bowel disease activity index (CIBDAI) scores [146]. In addition, in another group of dogs with chronic enteropathy, fecal calprotectin was correlated (r = 0.27) with disease activity and showed a high correlation (r = 0.9) with fecal S100A12 [147].

### 5.2. Cats

In cats, the role of calgranulins in the pathogenesis of chronic inflammatory enteropathy and intestinal lymphoma has been suggested [148]. In addition, fecal S100A12 concentrations at the time of diagnosis were higher in cats with chronic inflammatory enteropathy (CIE) and alimentary lymphoma (LSA) than in healthy controls, but did not differ between cats with LSA and those with CIE [148].

### 5.3. Porcine

In pigs, calprotectin expression in jejunal mucosa was decreased after treatment and tended to reduce intestinal inflammation in weaned pigs challenged with enterotoxigenic *Escherichia coli* (*E. coli*) [149]. Furthermore, fecal calprotectin levels in pigs were increased following the development of colitis, but do not significantly change due to enteritis [123]. 

### 5.4. Equine

In horses, serum calprotectin concentration was increased in animals with systemic inflammation produced by large colon ischemia and reperfusion [150]. Calprotectin expression in the colon was also increased in a case of colon inflammation after black walnut extract administration, which is associated with a systemic inflammatory response [151]. In equine saliva, calprotectin had a higher concentration in horses with equine gastric ulcer syndrome (EGUS) compared with healthy horses, although the concentration did not allow differentiation of horses with EGUS from horses with similar clinical signs due to other gastrointestinal causes [121].

### 5.5. Ruminants

In bovines, increases in serum calprotectin concentrations have been detected in calves with diarrhea caused by coronavirus but not in diarrhea caused by *Escherichia coli* or healthy calves [152].

### 5.6. Avian

Calprotectin in the serum and feces of broiler chickens has been described with a potential for the detection of low-grade chronic intestinal inflammation, which has a negative impact on production by the decrease in nutrient absorption in this species [153].

## 6. Inflammation and Sepsis in Animals

The S100 proteins have been involved in the following inflammatory conditions in animals.

### 6.1. Glomerulonephritis

Serum concentrations of S100A8/A9 are increased in mice with glomerulonephritis, whereas mice deficient in this protein were protected from this disease. This protein can exert and amplify inflammation through its interaction with different renal cells, and its blockade could be a therapeutic target in glomerulonephritis [154].

### 6.2. Lung Inflammation

In a mouse model of tuberculosis, it was demonstrated that the exacerbated lung inflammation of this disease is dependent on S100A8/A9 protein; which induces the production of proinflammatory cytokines and neutrophilic accumulation. These authors indicate that targeting S100A8/A9 has the potential to decrease lung tissue damage in this pathology [155]. In addition, in calves with pneumonia, S100A9 was increased in serum due to the associated systemic inflammatory response and also in BALF in which was a useful biomarker of pulmonary inflammation and damage [156].

### 6.3. Liver Inflammation

Using a mouse model of malaria, it was demonstrated that S100A9 is one of the key molecules involved in liver inflammation that is associated with this disease [157].

### 6.4. Sepsis

In a pilot study, serum calprotectin and S100A12 were higher in dogs with sepsis and dogs with systemic inflammatory response syndrome (SIRS) compared to healthy controls. In this study, the values of these analytes at the time of hospital admission did not differentiate between dogs with sepsis and SIRS, although they showed a different evolution in both processes during the following 24–48 h [102]. In pigs, calprotectin in saliva has been recently found to be increased in experimentally induced sepsis by *E. coli* lipopolysaccharide (LPS) administration, with higher increases found than in the saliva of pigs observed to have a non-septic inflammation [120].

## 7. Immunomediated Diseases in Animals

Calgranulins are increased in various immunomediated diseases, both naturally occurring and experimentally induced.

### 7.1. Atopic Dermatitis

Dogs with this disease show significantly higher serum concentrations of S100A8 than healthy dogs [158]. Similar findings have been described in humans in whom S100A8 and S100A9 increase in expression in both injured skin and serum from patients with atopic dermatitis, with the expression correlating with disease severity. Interestingly, house dust mites triggered the expression of both proteins [159]. It is important to point out that many S100 proteins are expressed in the epidermis and keratinocytes in humans [144] and therefore they can have applications as biomarkers of skin disorders which may also be the case in animals.

### 7.2. Autoimmune Uveitis

Rats with experimentally induced autoimmune uveitis showed an increase in expression of S100A8 in the eye, and S100A8 blockade could be a therapeutic agent in this disease [160].

### 7.3. Arthritis Rheumatoid (RA)

In a mouse model in which osteoarthritis was induced, S100A8/A9 was elevated in the synovium [161]. Of relevance is that S100A89, released from the synovium upon inflammation in experimental synovitis in mice, is an important mediator of pain response in the knee during the acute phase of inflammation [162]. Furthermore, in mice with experimentally induced arthritis, serum levels of S100A8/A9 were significantly increased and correlated with macroscopic joint swelling and histological inflammation, while serum levels of pro-inflammatory cytokines did not correlate with joint swelling. In addition, early serum S100A8/A9 levels were prognostic for disease outcomes at a later stage [163].

## 8. Obesity and Endocrine Disorders in Animals

In a report on human and mice obesity, serum calprotectin correlated with the visceral and subcutaneous fat area and body mass index in men, and S100A8 and S100A9 were overexpressed in adipose tissue in mice. This indicates that possibly adipocytes can be involved in the production of these S100 proteins, which could be considered potential markers of subclinical inflammation associated with obesity [164].

In miniature Schnauzers with idiopathic hyperlipidemia, there was an increase in serum calprotectin and S100A12, with the increase in serum calprotectin being associated with hyperlipidemia [165]. In dogs with diabetes mellitus, S100A12 was one of the proteins that showed a higher expression in saliva compared to healthy dogs [166].

In addition, in some endocrinopathies such as diabetes mellitus, a rat model showed that an increase in calprotectin in serum can indicate complications such as ischemic colitis [167].

Overall, this is really an area in which additional studies are needed in order to elucidate the role of calgranulins in obesity, insulin resistance and endocrine diseases in animals, and their possible use as biomarkers.

## 9. Conclusions

Calgranulins (S100A8, A9, and A12) are related to the activation of inflammation and the immune system and could be potentially used as biomarkers of inflammatory conditions as a complement to other traditionally used markers, such as white blood cell total and differential counts and morphological evaluation, as well as acute phase proteins or interleukin determination.

Previous reports have indicated that these proteins can increase during gastrointestinal diseases, inflammation and sepsis, immunomediated disorders, obesity, and endocrine disorders in different animal species. Further studies should help to clarify the possible practical applications of calgranulins as biomarkers for different disease conditions and species. Their assay in serum could be of potential use in some clinical aspects; for example, they could aid or improve the diagnosis and evaluation of the severity of a disease, inform whether the disease is active or not, detect inflammatory conditions, and also provide information about treatment monitoring and prognosis.

In addition, future studies could help to clarify the role and physiopathology of calgranulins in different diseases and evaluate their use as therapeutic targets, which could be of great interest in the veterinary field. For example, in humans, the administration of S100 antibodies or the use of therapeutic drugs to inhibit the action of S100s and therefore the induction of inflammation has been suggested for anti-inflammatory therapies.

Finally, the possibility of their measurement in non-invasive samples such as saliva or feces could enhance the application of assays for the calgranulins in animal health assessment. Further studies will help to improve basic biological knowledge of the S100 proteins as well as their clinical applications as biomarkers, which have a really promising future in the veterinary field.

## Figures and Tables

**Table 1:** S100 proteins, names, human diseases in which they have been studied, general reviews published, and veterinary studies (when available). There are 23 proteins in the table. S100A17 and S100A18 are also described, giving a total of 25 proteins in the group of S100 [5], but they are not included in this table since, to the authors’ knowledge, their functions and roles in diseases are still not well understood.

S100 Protein (Alternative Name)	Examples of Diseases in Which It Is Involved in Humans	General Reviews, Examples of Veterinary Studies
S100A1 (S100 alpha)	Neuroinflammation, Alzheimer’s disease [8] Vascular function and hypertension [9] Cardiotoxicity [10]	Structure, function, and therapeutic potential [11] Role in cardiovascular health and disease [12]
S100A2 (S100L, CAN19)	Endometrial carcinoma [13] Pancreatic cancer [14] Lung cancer [15] Keratinocyte damage in response to any inflammatory or toxic condition [16]	Influence in human diseases [17]
S100A3 (S100E)	Colorectal cancer [18] Pulmonary fibrosis [19]	
S100A4 (Metastasin1, Calvasculin)	Prostatic cancer [20] Rheumatoid arthritis [21]	Cancer progression and metastasis [22] Pathophysiology processes such as fibrosis, inflammation, immune response, neuroprotection, angiogenesis [23] Role in health and disease [24] ***Veterinary studies:*** Canine mammary carcinomas [25] Expressed in canine melanoma [26]
S100A5 (S100D)	Bladder cancer [27] Meningioma [28]	
S100A6 [Calcyclin (CACY)]	Osteosarcoma [29] Pancreatic cancer [30] Alzheimer’s disease [31]	Potential therapeutic target for acute myocardial infarction [32] Neurodegenerative diseases [33] ***Veterinary studies:*** Porcine reproductive and respiratory syndrome virus [34] Atopic dogs [35] *Toxoplasma gondii* infection [36]
S100A7 (Psoriasin1)	Psoriasis and inflammatory skin diseases [37] Squamous cell carcinomas and large cell lung carcinomas [38] Alzheimer disease [39] Wound healing process [40]	Role in breast cancer [41] ***Veterinary studies:*** Mastitis in goat [42]
S100A8 (Calgranulin A) S100A9 (Calgraunlin B) S100A8/A9 (Calprotectin)	Gastrointestinal disease, inflammation, autoimmune and septic conditions, among others. *See details for specific diseases in humans and veterinary in the text.*	Use of fecal calprotectin [43] Biological function and use as biomarker [44] Diagnosis of sepsis [45]
S100A10 (Calpactin-1 Plasminogen receptor S100A10)	Breast cancer [46]	Role in disease [47] Depression and antidepressant actions [48] Oncogenesis [49]
S100A11 (Calgizzarin S100C)	Reumatoid arthritis [50] Muscle damage and autoimmune disease [51] Ovarian cancer [52]	Role in disease [53]
S100A12 (Calgranulin C)	Inflammation, autoimmune and septic conditions. *See details for specific diseases in humans and veterinary in the text*	Digestive diseases [54] Atherosclerosis [55] Inflammation [56]
S100A13	Lung cancer [57] Melanoma [58] Thyroid cancer [59]	
S100A14	Breast cancer [60] Gastric cancer [61] Hepatocellular carcinoma [62]	Human cancer [63]
S100A15 (Koebnerisin)	Psoriasis [64] Hidradenitis suppurativa [65] Lung adenocarcinoma [66]	
S100A16	Breast Cancer [60] Lung adenocarcinoma [67] Gastric cancer [68] Insulin sensitivity [69]	Digestive system [70]
S100B		General diseases [71] Neurological disorders [72] Traumatic brain injuries [73] Sport-related concussion [74] ***Veterinary studies**:* Presence in milk of various species [75] Neurological diseases in calves [76]
S100G (Calbindin-DK9)	Pancreatitis [77]	
S100P (S100E)	Melanoma [78] Prostatic cancer [79]	Cancer [80]
S100Z	Liver cancer (with S100G) [81]	
**Others** Repetin Filaggrin Trichohyalin	Atopic dermatitis [82] Atopic dermatitis [83] Dermatological disorders [84]	

## Data Availability

Not applicable.
